# Efficacy of 20 min yoga module for reducing burnout among healthcare workers: protocol of randomised control trial and results of pilot study

**DOI:** 10.1136/bmjsem-2025-002637

**Published:** 2025-06-03

**Authors:** Vikas Upadhyay, Vartika Saxena, Apar Avinash Saoji, Monika Pathania, Bela Goyal

**Affiliations:** 1 All India Institute of Medical Sciences, Rishikesh, Uttarakhand, India; 2 Swami Vivekananda Yoga Anusandhana Samsthana, Bengaluru, Karnataka, India

**Keywords:** Stress, Chronic, Health promotion, Exercise, Anxiety

## Abstract

Burnout among healthcare workers is a significant global concern that affects their well-being and professional efficacy. Yoga has shown promise in reducing burnout and improving mental health outcomes. However, due to their hectic schedules, healthcare workers often struggle to find time for self-care. A 20 min yoga module has been developed specifically for them to address this. So, this study aims to assess the efficacy of a specific 20 min yoga module in reducing burnout among healthcare workers. An open-label, two-arm, randomised controlled trial involving healthcare workers aged 20–35 years participating for 4 weeks. Exclusion criteria included recent illness, respiratory ailments, pregnancy, life-threatening medical conditions or physical inability to perform yoga. After randomisation of 108 participants, they will be equally allocated to either the yoga group (20 min yoga) or the control group (20 min medium-paced walking). The primary outcome will be burnout, whereas the secondary outcomes include stress, anxiety, selective attention and happiness. Data collection: at baseline and after 4 weeks of intervention, compliance is monitored via daily attendance records. Descriptive and inferential analyses will employ intention-to-treat and per-protocol analysis using SPSS 26.0. A study with 20 participants found that a 20 min yoga intervention significantly improved emotional exhaustion, depersonalisation, personal accomplishment, happiness and Spielberger’s State-Trait Anxiety/Six-letter cancellation test scores. Cortisol levels showed trends of reduction but were not significant. These results inform the upcoming main trial. Ethical approval is obtained from the Institutional Ethical Committee via letter number AIIMS/IEC/20/762. The trial findings will be shared through peer-reviewed publications and presentations at conferences. Trial registration number: CTRI/2021/01/030568; Clinical Trial Registry of India.

**WHAT IS ALREADY KNOWN ON THIS TOPIC**

- Burnout, characterised by emotional, physical and mental exhaustion, is a significant concern among healthcare workers, with global rates of around 17.3% and higher rates in regions like India. Yoga, particularly pranayama and meditation, has been shown to reduce stress and burnout among healthcare professionals.

**WHAT THIS STUDY ADDS**

- This study assesses the efficacy of a 20 min yoga module specifically designed to reduce burnout among healthcare workers, aiming to provide a practical and accessible intervention that can be integrated into their demanding schedules.

**HOW THIS STUDY MIGHT AFFECT RESEARCH, PRACTICE OR POLICY**

- The findings could inform future research on short, targeted yoga interventions for healthcare workers and potentially influence workplace wellness programmes, offering a feasible solution to address burnout in the healthcare sector.

## Introduction

 Burnout, characterised by emotional, physical and mental exhaustion, stems from chronic workplace stress, according to the International Classification of Diseases−11. Its symptoms include feelings of exhaustion, detachment from one’s job and reduced professional efficacy.^1^ Burnout is a significant concern among healthcare workers worldwide, affecting approximately 17.3% globally and even higher rates in specific regions, such as India, where work-related burnout affects 26.9% of individuals, escalating to 52.8% during the pandemic.^2 3^ These professionals, who span various roles from direct patient care to healthcare management, are susceptible to burnout due to the demanding nature of their work.

Recognising the need to address burnout among healthcare workers, studies have explored alternative interventions such as yoga. Yoga, which incorporates physical postures (asana), controlled breathing (pranayama) and meditation (dhyana), has been shown to improve both physical and mental well-being.^4^ Specifically, pranayama techniques enhance the parasympathetic nervous system, potentially reducing mental stress.^5 6^ The combination of pranayama and meditation reduced burnout among healthcare workers, enabling them to cope with stress more effectively.

Studies have shown significant stress and burnout reduction among healthcare professionals through mind-body therapies such as yoga, which includes specific practices such as naadi-shuddhi pranayama, asana and gentle stretching.^7^ Nurses who participated in yoga for 6 months reported notably lower stress levels.^8^ Because healthcare workers may not have enough time for self-care, a specific 20 min yoga module was developed and validated to reduce burnout among healthcare workers.^9^

Building on existing research, this study aims to assess the efficacy of a 4-week, 20 min yoga module in reducing burnout among healthcare workers. By focusing on a concise and accessible yoga intervention, this study seeks to provide insights into the practicality and effectiveness of integrating yoga into the busy schedules of healthcare professionals. This approach offers a feasible solution for mitigating burnout in this essential workforce.

## Methods

### Trial design

**Figure 1:**
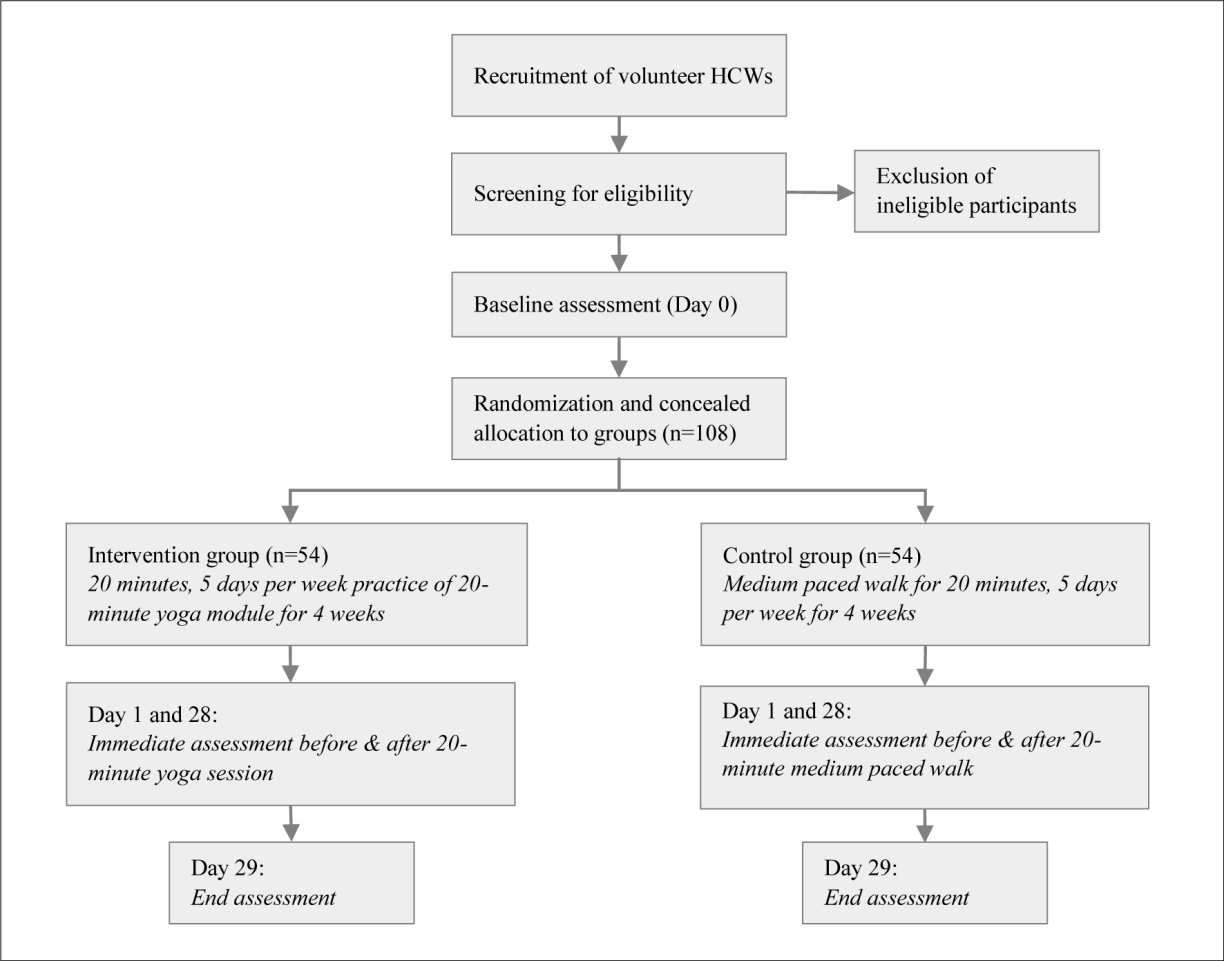
Trial design. HCWs, healthcare workers.

This will be an open-label, two-arm, randomised, controlled trial. The trial design is illustrated in figure 1. This trial follows the guidelines outlined in the Consolidated Standards of Reporting Trials statement.^10^ Table 1 presents the schedule of participant enrolment, time points and the variables assessed during each period per the Standard Protocol Items: Recommendations for Interventional Trials guidelines, which define standard protocol items for clinical trials.^11^

**Table 1:** Schedule of enrolment, intervention and assessments as per Standard Protocol Items: Recommendations for International Trials (SPIRIT)

	Study period					
	Enrolment	Allocation	Post-allocation			Close-out
Timepoint**	**−t_1_**	**0**	**t_0_**	**t_1_**	**t_2_**	**t_3_**
Enrolment:						
Eligibility screen	✔					
Informed consent	✔					
Allocation		✔				
Interventions:						
20 min yoga module				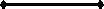		
20 min walking				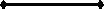		
Assessments:						
Socio-demographic details			✔			
Salivary cortisol, MBI, Happiness			✔			✔
STAI, SLCT				✔	✔	

.MBI, Maslach Burnout Inventory; SLCT, Six Letter Cancellation Test; STAI, State Trait Anxiety Inventory; t_0_, baseline; t_1_, day 1; -t1, enrolment; t_2_, day 28; t_3_, day 29.

### Participants

*The inclusion criteria are*: (1) healthcare worker age ranges from 20 to 35 years; (2) willingness to participate in the study for 4 weeks.

*The exclusion criteria* are as follows: (1) recent illness or surgery; (2) respiratory ailments (asthma, severe cough, cold, etc) refraining from breathing practices; (3) pregnant women; (4) any other life-threatening medical illness and (5) physical inability to perform the yoga module, which will not be included in the trial.

### Sample size

A sample size of 108 with 54 subjects in each arm (attrition rate 15%) is calculated based on an assumed effect size of 0.60 and with a type 1 error (α) of 0.05 and power (β) of 0.80, using the G Power software.^12^

### Sampling

All healthcare workers at AIIMS Rishikesh are included in the study frame. Invitations are being sent via email, departmental WhatsApp groups and physical flyers. Participants who expressed their willingness are contacted and recruited per eligibility criteria. They are provided with information about the study, answered their questions and given written details about the study. The principal investigator obtains informed consent before enrolment and baseline assessments. The recruitment process of the pilot study was started on 1 August 2022 and completed on 30 September 2022. The recruitment process for the main trial began on 1 May 2024 and is ongoing.

### Randomisation

After baseline assessments, participants will be randomly assigned in a 1:1 ratio to either the yoga group (20 min yoga) or the control group (20 min medium-paced walking). A random number sequence will be generated using a computer-generated programme in Microsoft Excel. The allocation sequence will be concealed in sequentially numbered, opaque, sealed envelopes. The corresponding author will generate random numbers and allocate sequences in opaque sealed envelopes.

### Treatments

#### Intervention group

The participants will be provided with a validated ‘20 min yoga module’^9^ comprising loosening exercises, scientific breathing techniques and meditative practices for 4 weeks. Brief details of the 20 min yoga module are presented in table 2.

**Table 2:** Components and steps of the 20 min yoga module

Serial number	Practices	Time duration (in min)
1	Loosening exercises	2
2	Breath awareness	1
3	Alternate nostril yoga breathing (round 1)	5
4	Rest	1
5	Alternate nostril yoga breathing (round 2)	5
6	AUM chanting	2
7	Humming bee breathing	3
8	Breath awareness	1
	Total	20

#### Control group

20 min medium-pace walking with 1600–2400 steps in 20 min, in a day for 4 weeks, will be provided as an intervention for reducing burnout among participants of this group. This walking intervention is metabolically matched to ensure comparability with other physical activity interventions, making it a suitable comparator. To check adherence and establish equality, all participants will download the free health app Google Fit.^13^ The Google Fit app will monitor the steps taken and walking duration.

A description of the interventions using the Template for Intervention Description and Replication checklist is provided in table 3.

**Table 3:** Description of interventions using the TIDieR checklist

Brief name	20 min yoga module’^9^ for the yoga group and 20 min medium-pace walking for the control group
Why	The 20 min yoga module helps reduce burnout and stress among healthcare workers by promoting relaxation through simple movements, breathing and mindfulness. It provides a quick break during busy shifts, easing tension, boosting focus and improving overall well-being. Regular practice can support long-term stress relief and better mental health.
What materials and procedures	Participants in the intervention group will attend 20 min offline yoga sessions 5 days a week for 4 weeks at AIIMS Rishikesh’s public health lab. The control group will engage in medium-paced walking for 20 min 5 days a week, aiming for 1600–2400 steps per session. They will use the Google Fit app to track their steps, distance and duration and submit daily screenshots of the data.
Who provided	The researcher, a certified yoga instructor, will take the yoga sessions, while participants in the control group will practice medium-paced walking independently, with monitoring by the researcher.
How	Participants in the intervention group will attend offline yoga sessions, while those in the control group will engage in medium-paced walking at their convenience.
Where	Offline yoga sessions will occur at the Public Health Lab of the Department of Community & Family Medicine, AIIMS Rishikesh. At the same time, medium-paced walking can be done at home or the workplace, depending on the participant’s convenience.
When and how much	Yoga sessions and medium-paced walking will be performed for 20 min 5 days a week over 4 weeks.
Tailoring	The 20 min yoga module will be adapted to each participant’s capabilities. Similarly, participants in the control group can walk 1600–2400 steps at a medium pace, based on their capability.

TIDieR, Template for Intervention Description and Replication.

### Outcomes

The primary outcome of this study is to compare the changes in burnout among healthcare workers after the daily practice of a 20 min yoga module to walking for a similar duration for 4 weeks. Secondary outcomes are changes in stress levels, anxiety, selective attention, and happiness scores among healthcare workers after the same 4-week intervention.

### Study tools

1. Demographic profile: participants’ names, age, sex and other socioeconomic indicators will be recorded.
2. Maslach Burnout Inventory (MBI) will be used to measure burnout. MBI is a 22-item survey that covers three areas: emotional exhaustion (EE), depersonalisation (DP) and personal accomplishment (PA) and uses a 7-point scale for responses. An answer can range from ‘never’ to ‘every day’. All three subscales show high internal consistency, with Cronbach’s α coefficients of 0.85, 0.76 and 0.71.^14^
3. Salivary cortisol levels will be measured using the Human Cortisol ELISA Kit (Diagnostic Biochem, Canada). Saliva samples will be collected using the SpeciMAX Saliva Collection Kit at the start, middle and end of duty hours, before (day 0) and after (day 29) intervention. The samples will be centrifuged at 4000 rpm and stored at −20°C before analysis. For extraction, 100 µL of cortisol-HRP conjugate and wash buffer will be added to 50 µL of each sample. The plates will be shaken at room temperature for 45 min, washed and incubated with the TMB substrate for 15–20 min. The reaction will be stopped with the solution, and the absorbance will be measured at 450 nm using a microplate ELISA reader. Cortisol extraction will follow the method described by Kirschbaum *et al*.^15 16^
4. Spielberger’s State-Trait Anxiety (STAI) Inventory will be used to measure state anxiety. STAI is a psychological inventory based on a 4-point Likert scale comprising 40 self-report questions. The STAI measures two types of anxiety—state anxiety, or anxiety about an event, and trait anxiety or anxiety as a personal characteristic. Higher scores are positively correlated with higher levels of anxiety.^17^ A high degree of internal consistency was observed for each of the 40 items, with Cronbach’s alpha value=0.38 to 0.89, while Cronbach’s alpha for the total scores is 0.86.^18^
5. Six-letter cancellation test (SLCT) for the measurement of selective attention. The SLCT consists of a sheet of 22 rows×14 columns of randomly arranged alphabet letters. The top of each sheet names six target letters. Participants will choose two possible strategies to cancel target letters: (1) all six letters at once or (2) selecting a single target letter at a time. It also suggests that, according to their choice, they follow horizontal, vertical or random paths on the test sheet. They will be asked to cancel as many target letters as possible during a test time of 90. Different randomised arrays of letters on the worksheet are used to compensate for memory effects. SLCT test–retest reliability was found (r=0.781, p=0.002).^19^ This test is directly related to attention measurements. This test has been used in earlier studies on the Indian population.^20^,^22^ Therefore, this test was validated in this study.
6. The self-rated scale of happiness will be used to measure happiness. A single self-rating scale will be used to assess happiness based on the question: ‘Do you feel happy in general?’. Following this question, a series of numbers from 0 to 10 will be written horizontally on one line with equal intervals. The research participants will be instructed: (a) imagine their global estimation and general feelings (not their present states), (b) take note that 0 is the minimum, 10 is the maximum score and (c) circle a number that best seems to describe their feelings. The 1-week test–retest reliability of the single-item self-rated happiness scale is 0.86.^23^

### Adverse events

The Institutional Ethics Committee (IEC) will be informed of any unexpected or serious adverse events within 48 hours of their occurrence. If any unexpected or serious adverse event occurs, participants will be provided medical treatment immediately.

### Analysis of outcomes

Intention-to-treat (ITT) and per-protocol analyses will be performed to provide a comprehensive understanding of intervention effects. The proportions and means will be calculated for descriptive analysis. The normality of the data will be checked using the Shapiro-Wilk test. Continuous data will be reported as mean±SD and compared using an independent t-test. Categorical data will be reported as frequencies and compared using the χ^2^ test. An independent t-test will compare pre and postintervention data of all group outcomes. A paired t-test will be used to compare the pre and postdata of all outcomes within the group. Statisticians will be blinded to group assignments for statistical analysis. Statistical calculations will be performed by SPSS V.26.0.

### Data collection and follow-up

Data will be collected at baseline and after fourth week. The researcher will take yoga classes at the Public Health Lab of the Department of Community and Family Medicine, and compliance will be monitored by recording the daily attendance of participants. If a subject cannot attend at least 4 days a week, he/she will not be included in the analysis.

## Results of the pilot study

A pilot study was conducted to evaluate the feasibility of an intervention. The quality of practice was assessed using a 10-point Likert visual analogue scale, with participants achieving an average mean score of 9 out of 10. This high score indicates strong acceptability among participants, demonstrating their comfort and ability to perform the practices included in the yoga module effectively.

A total of 20 participants were recruited for the pilot study, and informed consent was obtained from each participant. The participants, with a mean age of 31.6±5.3 years, were randomly assigned to either the yoga group (20 min yoga session) or the control group (20 min medium-paced walking). The interventions were delivered in offline classes and conducted at least 5 days a week over 4 consecutive weeks. Burnout scores were measured using the MBI, salivary cortisol levels were assessed with the ELISA technique and happiness scores were assessed using a self-rated happiness scale at baseline and after the 4-week intervention. State anxiety and selective attention were assessed using the STAI and SLCT, respectively, before and after the 20 min intervention at both baseline and after the 4 week intervention. Normality was checked with the Shapiro-Wilk test. Paired and independent t-tests were used for normally distributed data, while the Wilcoxon Signed Rank and Mann-Whitney U tests were used for non-normally distributed data.

Seventeen participants (nine from the yoga group and eight from the control group) completed the intervention. Statistical tests revealed a statistically significant improvement in scores in all the domains of burnout in the yoga group compared with baseline, as shown in table 4. Significant changes were found in EE (p=0.009), DP (p=0.028), and PA (p=0.03) in the yoga group. In contrast, no significant changes were observed in the control group across these domains: EE (p=0.31), DP (p=0.28), and PA (p=0.68).

**Table 4:** Pre and postmean scores of MBI, salivary cortisol, happiness scale, STAI and SLCT in healthcare workers after 4 weeks of intervention

			Yoga group	Control group	P value
MBI					
Emotional exhaustion	Day 0	Pre	24.67±7.05	22.75±8.81	0.631*
	Day 29	Post	19.44±7.09	21±9.18	0.705*
		P value	0.009†	0.31†	
Depersonalisation	Day 0	Pre	7±5.72	5.5±2.98	0.481‡
	Day 29	Post	4.33±3.77	4±3.42	0.423‡
		P value	0.028§	0.324§	
Personal accomplishment	Day 0	Pre	26.56±8.08	24.5±12.92	0.705*
	Day 29	Post	29.56±8.68	26±12.2	0.506*
		P value	0.037†	0.683†	
Salivary cortisol					
Concentration (ng/mL)	Day 0	Pre	77.89±17.5	71±20.97	0.477*
	Day 29	Post	63.11±25.07	64.13±26.58	0.937*
		P value	0.078†	0.486†	
Happiness scale					
Scores	Day 0	Pre	7.5±1.04	7.8±0.8	0.579*
	Day 29	Post	8.5±1.04	8±1.2	0.218*
		P value	0.007†	0.197†	
STAI					
Scores	Day 1	Pre	35.89±4.05	34.59±3.14	0.469*
		Post	32.66±3.16	34.76±3.33	0.205*
		P value	0.071†	0.922†	
	Day 28	Pre	38.79±5.23	32.04±7.25	0.049*
		Post	30.9±3.28	29.51±5.92	0.566*
		P value	0.017†	0.154†	
SLCT					
Scores	Day 1	Pre	25.89±6.58	26.54±4.89	0.82*
		Post	31.78±7.55	25.06±2.62	0.031*
		P value	0.021†	0.418†	
	Day 28	Pre	32.78±7.6	28.88±8.51	0.338*
		Post	38.67±9.49	30.38±6.95	0.057*
		P value	0.001†	0.255†	

P < 0.05 considered statistically significant.

* Independent t test.

† Paired t test.

‡ Mann-Whitney U test.

§ Wilcoxon Signed Rank Test.

MBI, Maslach Burnout Inventory; SLCT, Six Letter Cancellation Test; STAI, State Trait Anxiety Inventory.

The yoga group showed a trend towards reduced salivary cortisol levels (14.78±21.96 ng/mL), but this change did not reach statistical significance (p=0.078). The control group experienced a smaller decrease in salivary cortisol levels (6.87±26.43 ng/mL), which also did not reach statistical significance (p=0.48), as shown in table 4. Additionally, the yoga group had a significant (p=0.007) increase in happiness scores. Mean pre–post scores of the happiness scale are shown in table 4.

Significant changes were observed in the yoga group across both STAI and SLCT scores following the intervention. On day 1, while the Yoga group showed a non-significant (p=0.071) decrease in STAI scores, there was a significant (p=0.021) improvement in SLCT scores. On day 28, while the Yoga group showed a significant reduction in STAI scores (p=0.017), there was a substantial improvement in SLCT scores (p=0.001). In contrast, the control group showed no significant changes in either STAI or SLCT scores at any time. The mean scores of STAI and SLCT on day 1 and day 28, immediately before and after the intervention, are shown in table 4.

In conclusion, the 20 min yoga intervention significantly improved burnout scores, particularly in EE, DP and PA. While trends towards reduced salivary cortisol levels were noted in the yoga group, these findings did not reach statistical significance. These results will inform the main trial that follows.

## Discussion

This is the first trial to assess the efficacy of a 20 min yoga module in reducing burnout among healthcare workers. The findings of this study contribute to the growing body of evidence supporting the effectiveness of yoga as a preventive and therapeutic intervention for burnout in healthcare settings.

The intervention incorporates components such as loosening exercises (*sukshma vyayama*), regulated breathing techniques (*pranayama*) and meditation (*dhyana*). These practices are designed to address various dimensions of burnout while also exploring secondary outcomes like stress reduction, anxiety management, selective attention and happiness. Previous research supports the effectiveness of yoga-based interventions in improving professional quality of life and reducing burnout. For instance, a randomised controlled trial conducted at a tertiary care hospital demonstrated significant improvements in EE, DP and PA among healthcare providers following a 12-week yoga-based programme (p<0.001)2.^24^ Similarly, workplace yoga programmes have reduced fatigue and burnout among blue-collar workers, highlighting their feasibility and utility across diverse occupational settings.^25^

A systematic review further corroborates the positive impact of yoga on stress management among healthcare workers, with studies reporting improvements in self-regulation, self-compassion and stress levels following yoga interventions.^26^ Additionally, the development of shorter modules, like the 20 min intervention used in this study, addresses practical barriers such as time constraints faced by healthcare workers.^9^ These findings collectively underscore the potential of yoga to enhance resilience, emotional control and cognitive functioning while fostering a sense of PA and fulfilment.

By examining primary (burnout reduction) and secondary outcomes (eg, mindfulness and happiness), this study contributes to the growing body of evidence advocating for integrating accessible yoga practices into healthcare settings. Future research could explore long-term effects and broader applications across different populations to validate these promising results.
